# Altered Functional Connectivity and Small‐World Network Properties in MDD With Daytime Sleepiness: Implications for Anhedonia

**DOI:** 10.1155/da/9599586

**Published:** 2026-09-15

**Authors:** Yifei Li, Shukun Zhu, Qinghong Hu, Peng Fang, Yu Zhang, Jiajia Zhu, Dao-min Zhu

**Affiliations:** ^1^ Department of Sleep Disorders, Affiliated Psychological Hospital of Anhui Medical University, Hefei 230022, China; ^2^ Hefei Fourth People’s Hospital, Hefei 230022, China; ^3^ Anhui Mental Health Center, Hefei 230022, China, ahmhcentre.com; ^4^ The School of Mental Health and Psychological Sciences, Anhui Medical University, Hefei 230032, China, ahmu.edu.cn; ^5^ Department of Radiology, The First Affiliated Hospital of Anhui Medical University, Hefei, China, ahmu.edu.cn

**Keywords:** brain functional networks, functional magnetic resonance imaging, graph theory analysis, major depressive disorder, physical anhedonia, polysomnography

## Abstract

**Background:**

Approximately 37.4% to 57.1% of people with major depressive disorder (MDD) exhibit symptoms of daytime sleepiness (DS). Patients with MDD with concurrent DS tend to have worse clinical outcomes and often experience severe anhedonia. As a key symptom of MDD, anhedonia is strongly associated with its severity. The aim of this study was to clarify the neuroimaging mechanisms of DS symptoms in MDD patients with anhedonia.

**Methods:**

A total of 22 patients were recruited into the DS group (Epworth Sleepiness Scale [ESS] score ≥ 7), and 45 patients were included in the no DS group (No DS) in this cross‐sectional study. All subjects underwent resting state functional MRI, polysomnography (PSG) and clinical scale assessments. Anhedonia is assessed using the Revised Social Anhedonia Scale (RSAS), Revised Physical Anhedonia Scale (RPAS), and Temporal Experience of Pleasure Scale (TEPS). Employing the Dosenbach 160‐node atlas, we obtained whole‐brain functional networks and extracted topological features (global efficiency [Eglob], local efficiency [Eloc], path length [Lp], and clustering coefficient [Cp]) using a graph‐theoretic approach. Group comparisons, covariate‐adjusted regression analyses, and mediation analysis were performed to examine the association between ESS, anhedonia, and ventrolateral prefrontal cortex (vlPFC)–posterior cingulate cortex (PCC) functional connectivity (FC).

**Results:**

The two groups were comparable for demographic and clinical characteristics, with no differences in Hamilton Depression Rating Scale (HAMD) or Hamilton Anxiety Rating Scale (HAMA) scores or polysomnographic parameters. The DS group scored higher on the RPAS, while social and temporal anhedonia did not differ between groups. After adjusting for relevant clinical and medication covariates, continuous ESS scores remained independently associated with RPAS. In terms of brain networks, the DS group showed reduced FC between the default mode network (DMN) and frontoparietal network (FPN)—primarily linking the vlPFC with the PCC and precuneus—as well as within and between posterior parieto‐occipital and visual networks (VNs). The DS group also exhibited extensive reductions in Eglob, Eloc, and Cp, with prolonged Lp. Mediation analysis revealed that vlPFC–PCC FC significantly mediated the association between ESS and RPAS.

**Conclusion:**

MDD patients with DS exhibit more severe physical anhedonia, which may be associated with reduced indicators of their extensive whole‐brain networks and reduced FC between some functional brain networks. Our study may provide a neuroimaging mechanistic basis for clinical identification and intervention of anhedonia in MDD patients with DS.

## 1. Introduction

Daytime sleepiness (DS) is a common symptom in a variety of psychiatric disorders, particularly affective disorders [1]. However, assessing DS in affective patients can be challenging. Despite the frequent complaints of DS among individuals with affective disorders, objective test results, such as the multiple sleep latency test, often fall within the normal range [2]. Notably, among different types of affective illnesses, patients with major depressive disorder (MDD) report the highest prevalence of DS symptoms. MDD, a highly prevalent mental health condition, significantly affects psychosocial functioning and undermines the quality of life for those affected. The World Health Organization (WHO) predicts that by 2030, major depression will rank as the leading cause of disease burden worldwide [3]. Therefore, it is crucial to pay closer attention to the symptoms of DS in MDD patients.

Approximately 37.4% to 57.1% of patients with MDD exhibit DS symptoms [4–6]. These patients tend to experience more severe depressive symptoms, carry a higher risk of suicide, and generally face poorer clinical outcomes [7, 8]. Understanding the underlying neural mechanisms is therefore of urgent importance. Furthermore, individuals with DS are more likely to suffer from MDD, and DS symptoms often persist even after MDD treatment, correlating with poorer prognosis [9, 10]. Unfortunately, the neural mechanisms underlying DS in MDD patients remain inadequately explored. The multiple sleep latency test has yet to effectively identify DS symptoms in most MDD patients [11].

Resting‐state functional magnetic resonance imaging (rs‐fMRI) is a neuroimaging technique used to assess the functional connectivity (FC) between brain regions when subjects are not performing specific tasks [12]. Previous research has shown that adults experiencing DS have increased FC in executive and salience networks, with altered nodal topological properties in the sensory cortex, putamen, and anterior cingulate correlated with DS severity [13]. An fMRI study of Parkinson’s disease patients with DS reported increased FC in the default mode network (DMN), which was associated with their DS symptoms [14]. Another rs‐fMRI study found that FC between the thalamus and left striatum was reduced in MDD patients with DS and significantly associated with DS symptoms [15]. Despite these findings, there is a lack of fMRI studies specifically examining anhedonia in MDD patients with DS. Previous work by our group has demonstrated that MDD patients with DS experience more severe anhedonia, but we have not yet investigated the underlying neuroimaging mechanisms [16]. Given that anhedonia is a core symptom of MDD and is closely linked to its severity and reflects dysfunction in cortico‐striatal reward circuitry [17], the present study aims to investigate the neuroimaging changes in MDD patients with DS symptoms and to test whether large‐scale brain network alterations mediate the previously reported association between DS and physical anhedonia in MDD. We thereby aim to address a significant gap in the literature and to enhance our understanding of the neural mechanisms underlying DS in MDD.

## 2. Materials and Methods

### 2.1. Participants

This was a single‐center analytical cross‐sectional observational study. A total of 67 patients with MDD aged 18–60 years were consecutively recruited from the Sleep Disorders Department of the Psychological Hospital of Anhui Medical University from 2023.08 to 2024.12. Inclusion criteria were as follows: (1) age 18–60 years, (2) ICD‐10 diagnosis of MDD by two independent trained psychiatrists, and (3) no other current Axis‐I psychiatric disorder. Exclusion criteria were as follows: (1) any history of serious neurological or systemic medical illness, (2) head injury with loss of consciousness, (3) current pregnancy or lactation, (4) MRI contraindications, (5) current substance use disorder, and (6) cognitive impairment precluding informed consent. All 67 enrolled participants completed the clinical, polysomnographic, and MRI assessments; met the imaging quality–control criteria; and were included in the final analyses. No a priori sample–size calculation was performed because this was an exploratory neuroimaging study; the sample comprised all eligible patients consecutively recruited during the predefined recruitment period. Detailed information on antidepressant (AD) and sedative–hypnotic use (drug class, daily dose, duration, and stability over the prior 2 weeks) was retrieved from medical records and is summarized in Table 1. To facilitate covariate adjustment, AD doses were converted to fluoxetine‐equivalent milligrams; sedative‐side‐effect ADs (mirtazapine, trazodone, mianserin, doxepin, and maprotiline) were tabulated separately as a sedating‐AD–equivalent dose.

**Table 1:** Demographic and clinical characteristics of DS and No DS groups.

Characteristics	DS group (*n* = 22)	No DS group (*n* = 45)	*t*/*x* ^2^	*p*
Age (year)	38.36 ± 12.27	37.71 ± 13.64	0.190	0.850
Gender (female)	13 (59.09%)	26 (57.78%)	*x* ^2^ = 0.01	0.918
Education (year)	12.57 ± 3.90	13.11 ± 3.89	−0.524	0.603
Total disease duration (year)	6.25 ± 8.79	5.69 ± 6.89	0.283	0.778
Current episode duration (months)	5.44 ± 12.39	7.91 ± 9.96	−0.881	0.381
HAMD	33.77 ± 7.68	34.80 ± 7.09	−0.542	0.590
HAMA	22.41 ± 7.81	21.13 ± 5.88	0.747	0.458
RSAS	21.41 ± 7.81	19.80 ± 6.17	0.917	0.362
RPAS	26.05 ± 8.32	20.87 ± 8.74	2.314	0.024
TEPS	52.55 ± 23.81	58.62 ± 16.87	−1.073	0.292
Sedative‐hypnotic use				
No	2 (9.1%)	2 (4.4%)	—	—
BZDs	9 (40.9%)	17 (37.8%)	—	—
NBZDs	10 (45.5%)	18 (40.0%)	—	—
BZDs and NBZDs	1 (4.5%)	8 (17.8%)	*x* ^2^ = 2.960	0.398
Antidepressant equivalent dose (fluoxetine‐eq, mg/day)	23.19 ± 14.19	18.92 ± 17.75	0.986	0.328
Sedating‐AD–equivalent dose (mg/day)	2.05 ± 3.67	1.98 ± 4.79	0.059	0.953
Mean framewise displacement (mm)	0.06 ± 0.03	0.06 ± 0.04	−0.253	0.801

*Note:* Comparisons of demographic and clinical characteristics between MDD patients with DS and those without DS. Data are presented as mean ± standard deviation or percentage. *t*‐Values are reported for continuous variables, and chi‐square (*x* ^2^) values are reported for categorical variables. *p*‐Values indicate the level of significance for between‐group differences.

Abbreviations: BZDs, benzodiazepines; HAMA, Hamilton Anxiety Rating Scale; HAMD, Hamilton Depression Rating Scale; NBZDs, nonbenzodiazepine sedatives; RPAS, Revised Physical Anhedonia Scale; RSAS, Revised Social Anhedonia Scale; TEPS, Temporal Experience of Pleasure Scale.

As part of the assessment, participants completed the Epworth Sleepiness Scale (ESS) [18], the Revised Social Anhedonia Scale (RSAS) [19], the Revised Physical Anhedonia Scale (RPAS) [20], and the Temporal Experience of Pleasure Scale (TEPS) [21]. A 24‐item Hamilton Depression Rating Scale (HAMD) scale [22] and a 14‐item Hamilton Anxiety Rating Scale (HAMA) scale [23] were also used to quantify depressive and anxiety symptoms for each participant.

### 2.2. Epworth Sleepiness Score

Subjects with ESS total scores ≥ 7 were classified as having clinically meaningful DS (DS group, *n* = 22); the remaining patients formed the No DS group (*n* = 45). Although ESS ≥ 10 is widely cited as an international cutoff, validation work in Chinese populations indicates that the mean ESS score in healthy Chinese adults is ~7.5 ± 3.0—substantially lower than that reported in Western cohorts [24]. Accordingly, an ESS score ≥ 7 in a Chinese MDD cohort already represents the upper boundary of the normal range and captures subclinical‐to‐mild DS that may carry neurobiological significance, an approach consistent with several previous Chinese psychiatric studies. To address potential concerns regarding this threshold, we additionally performed a sensitivity analysis using ESS ≥ 10 (Supporting Information), with all primary analyses repeated under both cutoffs.

### 2.3. Polysomnography (PSG) Examination

All patients underwent overnight PSG examinations using an Embla N7000 instrument (New York, USA), in accordance with the American Academy of Sleep Medicine (AASM) guidelines, with manual analysis. Participants were instructed to abstain from caffeine, tea, and alcohol during the recording period.

The PSG recordings included a comprehensive range of physiological parameters. Neurophysiological variables such as the electroencephalogram (EEG), electrooculogram (EOG), chin electromyogram (EMG), and lower extremity movement were recorded, along with cardiorespiratory variables, including electrocardiogram (ECG), thoracic and abdominal movements, oxygen saturation (SpO2), and oronasal airflow. Additionally, other relevant variables, such as body position, were also recorded throughout the study. All signals were automatically recorded and subsequently analyzed in detail.

From these recordings, the following variables were extracted: time in bed (TIB), total sleep time (TST), sleep latency, N1 duration, N1 percentage (N1%), N2 duration, N2 percentage (N2%), N3 duration, N3 percentage (N3%), apnea‐hypopnea index (AHI), and oxygen desaturation index (ODI). TIB was defined as the duration from “lights out” to “lights on,” while TST was calculated as the total amount of sleep achieved during both nonrapid eye movement (NREM) and rapid eye movement (REM) sleep periods.

### 2.4. Image Acquisition

MRI scans were performed using a 3.0 T MRI system equipped with a 24‐channel head coil (Discovery MR750 advance, General Electric Company, Milwaukee, Wisconsin, USA). To ensure participant comfort and minimize head motion, earplugs were provided to reduce scanner noise, and foam padding was used. Participants were queried during and after scanning to confirm that none had fallen asleep. All T1‐weighted structural images were visually inspected for any visible artifacts related to head movement. Similarly, all functional images were reviewed to confirm that the entire cerebellum was adequately covered. No participants were excluded based on visual inspection of imaging artifacts.

High‐resolution 3D T1‐weighted structural images were acquired using a brain volume (BRAVO) sequence with the following parameters: repetition time (TR) = 8.5 ms, echo time (TE) = 3.2 ms, inversion time (TI) = 450 ms, flip angle (FA) = 12°, field of view (FOV) = 256 mm × 256 mm, matrix = 256 × 256, slice thickness = 1 mm with no gap, 188 sagittal slices, and an acquisition time of 296 s.

Resting‐state blood‐oxygen‐level‐dependent (BOLD) data were acquired using a gradient‐echo single‐shot echo planar imaging (GRE‐SS‐EPI) sequence with the following parameters: TR = 2000 ms, TE = 30 ms, FA = 90°, FOV = 220 mm × 220 mm, matrix = 64 × 64, slice thickness = 3 mm, slice gap = 1 mm, 35 interleaved axial slices, 185 volumes, and an acquisition time of 370 s.

### 2.5. fMRI Preprocessing

Resting‐state BOLD images were preprocessed using DPABI (v8.0) [25] running on MATLAB. The preprocessing pipeline comprised (1) removal of the first 10 volumes to allow magnetization equilibrium; (2) slice‐timing correction; (3) head‐motion correction with realignment of all volumes to the first acquired volume; (4) coregistration of T1‐weighted images to functional volumes; (5) tissue segmentation and normalization to Montreal Neurological Institute (MNI) space using DARTEL, with resampling to 3 × 3 × 3 mm isotropic voxels; (6) spatial smoothing with a Gaussian kernel of 4 mm full width at half maximum (FWHM); (7) regression of nuisance signals, including 24 motion parameters (Friston model), white‐matter signal, and cerebrospinal‐fluid signal; and (8) band‐pass filtering (0.01–0.1 Hz). Mean framewise displacement (FD) was computed per subject using the Jenkinson formulation. No subject exhibited a maximum FD exceeding 2 mm; mean FD did not differ between the DS and No DS groups (DS: 0.058 ± 0.026 mm; No DS: 0.060 ± 0.037 mm; *t* = −0.25, *p* = 0.80). Mean FD was nonetheless included as a nuisance covariate in subsequent neuroimaging analyses, as appropriate.

### 2.6. Functional Brain Network Construction

A brain network consists of interconnected nodes and edges, with nodes identified using Dosenbach’s 160‐atlas [26]. The edges denote the FC between each node. For each node, a 5‐mm‐radius sphere was constructed centered at the coordinates specified by the atlas. The neural activity of each node was determined by averaging the preprocessed BOLD signals within the sphere. To create the whole brain’s connectivity matrix, Pearson correlation coefficients were calculated for all edges, followed by Fisher’s transformation to convert these coefficients into *z* values. Weighted topological properties of the FC matrices were assessed across various levels of network sparsity for each subject [27].

The Yeo atlas categorizes the human cerebral cortex into seven distinct networks based on functional parcellations derived from 1000 healthy individuals [28]. This study selected six of the seven Yeo networks: the dorsal attention network (DAN), DMN, somatomotor network (SMN), ventral attention network (VAN), visual network (VN), and frontoparietal network (FPN). The limbic network was excluded because no Dosenbach regions of interest (ROIs) were situated within it. Subcortical ROIs were designated as an independent “subcortical network,” serving as the seventh network. Finally, between‐ and within‐network FC was evaluated by averaging the corresponding *z*‐scores.

### 2.7. Network Analysis

Both global and nodal brain network metrics were determined at various sparsity thresholds using the Brain Connectivity Toolbox [29]. The global metrics included global efficiency (Eglob), local efficiency (Eloc), path length (Lp), and clustering coefficient (Cp). For each network metric, the area under the curve (AUC) across the entire range of sparsity thresholds was computed to provide a measurement with enhanced sensitivity [27].

For the neuroimaging data, group differences in large‐scale FC and the AUC of these global and nodal network metrics were assessed. To rigorously control for potential confounding effects, these neuroimaging comparisons were conducted using general linear models adjusted for covariates including age, sex, current episode duration, HAMD, sedative‐hypnotic use (binary), sedating‐AD–equivalent dose, and mean FD.

### 2.8. Statistical Analysis

Demographic and clinical data were analyzed using SPSS 25.0. Continuous variables (e.g., RPAS, RSAS, TEPS, HAMD, HAMA, AHI, sleep parameters, and equivalent medication doses) were compared between the DS and No DS groups using independent‐samples *t*‐tests, with Mann–Whitney *U* tests as nonparametric sensitivity analyses for variables violating normality (Shapiro–Wilk *p*  < 0.05) or homogeneity of variance (Levene’s *p*  < 0.05); categorical variables were compared using *χ* ^2^ tests or Fisher’s exact tests as appropriate. To assess the relationship between DS and anhedonia beyond the dichotomous group comparison, we performed (a) Spearman correlation between continuous ESS scores and the three anhedonia scales; (b) partial correlations controlling for age, sex, current episode duration, HAMD, HAMA, AHI, sedative‐hypnotic use, and sedating‐AD–equivalent dose; and (c) a hierarchical multiple linear regression was performed with the anhedonia score showing a significant association with ESS in the partial correlation analysis as the dependent variable, ESS as a continuous predictor, and the same covariates entered. Group comparisons of resting‐state FC and graph‐theoretical metrics (Cp, characteristic Lp, Eloc, Eglob, and AUC across sparsity thresholds) were performed in DPABI using two‐sample *t*‐tests, with age, sex, current episode duration, HAMD, sedative‐hypnotic use (binary), sedating‐AD–equivalent dose, and mean FD entered as nuisance covariates. Covariates were selected based on their potential associations with DS, anhedonia, or neuroimaging measures and, where applicable, to account for medication exposure and head‐motion effects. The false discovery rate (FDR) correction method was applied to control for multiple comparisons across nodes and functional edges. Sensitivity analyses using a minimally adjusted model (covariates: age, sex, and mean FD) are reported in the Supporting Information to facilitate comparison. Mediation analysis was conducted using the PROCESS macro (Hayes, Model 4) in SPSS 25.0 to test our a priori hypothesis that DS contributes to physical anhedonia via altered ventrolateral prefrontal cortex (vlPFC)–posterior cingulate cortex (PCC) FC. ESS was entered as the predictor (X), vlPFC–PCC FC as the mediator (M), and RPAS as the outcome (Y); age, sex, and mean FD were entered as covariates. The significance of the indirect effect was assessed using bias‐corrected (BC) bootstrapping with 5000 resamples to generate a 95% confidence interval (CI); a CI excluding zero was taken as evidence of a significant indirect effect. Two‐tailed *p*  < 0.05 was considered statistically significant unless otherwise specified. There were no missing data for variables included in the primary analyses; therefore, no imputation was performed. A schematic overview of the study workflow is provided in Figure S1.

## 3. Results

### 3.1. Comparisons Between All Patients With DS vs. No DS

There were no significant differences between the DS group and the No DS group in terms of sedative‐hypnotic drug use, education level, or total disease duration. However, RPAS scores were elevated in MDD patients with DS symptoms (Table 1). Individual‐level distributions of RPAS scores by group are shown in Figure S2. No significant differences were found in PSG parameters between the two groups (Table 2).

**Table 2:** Polysomnographic parameters of DS and No DS groups.

Characteristics	DS group (*n* = 22)	No DS group (*n* = 45)	*t*	*p*
TST	395.61 ± 120.27	423.33 ± 93.96	−1.032	0.306
TIB	518.20 ± 31.17	511.11 ± 27.55	0.948	0.347
Sleep latency	28.18 ± 17.93	36.47 ± 58.16	−0.651	0.517
N1 duration	58.10 ± 28.24	50.97 ± 30.89	0.913	0.365
N1%	0.14 ± 0.08	0.13 ± 0.10	0.542	0.590
N2 duration	211.82 ± 69.92	241.80 ± 78.33	−1.522	0.133
N2%	0.49 ± 0.14	0.55 ± 0.142	−1.815	0.074
N3 duration	80.81 ± 51.50	69.29 ± 51.43	0.861	0.392
N3%	0.18 ± 0.12	0.16 ± 0.11	1.024	0.310
AHI	3.48 ± 5.53	4.44 ± 6.95	−0.563	0.575
ODI	3.55 ± 5.58	4.50 ± 9.96	−0.557	0.580

*Note:* Comparisons of polysomnographic parameters between MDD patients with DS and those without DS. Data are presented as mean ± standard deviation. *t*‐Values are reported for continuous variables, and *p*‐Values indicate the level of significance for between‐group differences. N1, stage N1 sleep; N2, stage N2 sleep; N3, stage N3 sleep.

Abbreviations: AHI, apnea‐hypopnea index; ODI, oxygen desaturation index; TIB, time in bed; TST, total sleep time.

To complement the dichotomous group comparison, we examined the association between ESS (modeled as a continuous variable) and the three anhedonia scales. ESS was selectively associated with physical anhedonia (RPAS): Spearman *ρ* = 0.248, *p* = 0.043; partial *r* = 0.294, *p* = 0.024 (controlling for age, sex, current episode duration, HAMD, HAMA, AHI, sedative‐hypnotic use, and sedating‐AD dose). The corresponding Spearman partial correlation gave nearly identical results (*ρ* = 0.287, *p* = 0.028). No significant associations were observed between ESS and social anhedonia (RSAS, partial *R* = 0.075, *p* = 0.573) or temporal pleasure (TEPS, partial *R* = −0.146, *p* = 0.269) (Table 3). Because RPAS was the only anhedonia measure showing a significant association with ESS in the partial correlation analyses, we conducted a follow‐up hierarchical multiple regression for RPAS only. After adjusting for all covariates, ESS remained an independent predictor of RPAS (standardized *β* = 0.282, *t* = 2.32, *p* = 0.024); current episode duration and AHI also independently predicted RPAS (*β* = 0.276, *p* = 0.030; and *β* = −0.277, *p* = 0.046) (Table 4). Variance inflation factors (VIFs) were < 1.55 for all predictors, indicating no multicollinearity.

**Table 3:** Spearman and partial correlations between ESS and anhedonia scales in patients with MDD.

Outcome	Spearman *ρ*	*p*	Partial *R* ^a^	*p*
RPAS	0.248	0.043 ^∗^	0.294	0.024 ^∗^
RSAS	−0.000	0.999	0.075	0.573
TEPS	−0.056	0.654	−0.146	0.269

*Note:* Spearman *ρ* represents the unadjusted rank–order correlation between continuous ESS scores and each anhedonia scale.

Abbreviations: ESS, Epworth Sleepiness Scale; RPAS, Revised Physical Anhedonia Scale; RSAS, Revised Social Anhedonia Scale; TEPS, Temporal Experience of Pleasure Scale.

^a^Partial *R* represents the Pearson partial correlation adjusted for age, sex, current episode duration, HAMD, HAMA, AHI, sedative‐hypnotic use, and sedating‐antidepressant–equivalent dose.

^∗^ *p* < 0.05.

**Table 4:** Multiple linear regression predicting physical anhedonia (RPAS) severity.

Predictor	*B*	SE	*β*	*p*	VIF
ESS	0.530	0.228	0.282	0.024 ^∗^	1.10
Age	−0.045	0.092	−0.067	0.623	1.37
Sex (female)	2.163	2.418	0.121	0.375	1.36
Current episode duration (months)	0.227	0.102	0.276	0.030 ^∗^	1.14
HAMD	−0.165	0.163	−0.134	0.317	1.32
HAMA	−0.045	0.194	−0.033	0.819	1.52
AHI	−0.380	0.186	−0.277	0.046 ^∗^	1.37
Sedative‐hypnotic use	1.869	4.590	0.050	0.685	1.13
Sedating‐AD–equivalent dose	−0.147	0.250	−0.073	0.558	1.15

*Note:* B, unstandardized regression coefficient; *β*, standardized regression coefficient.

Abbreviations: AD, antidepressant; AHI, apnea‐hypopnea index; ESS, Epworth Sleepiness Scale; HAMA, Hamilton Anxiety Rating Scale; HAMD, Hamilton Depression Rating Scale; RPAS, Revised Physical Anhedonia Scale; SE, standard error; VIF, variance inflation factor.

^∗^ *p*  < 0.05.

### 3.2. Large‐Scale Network FC Comparison

**Figure 1:**
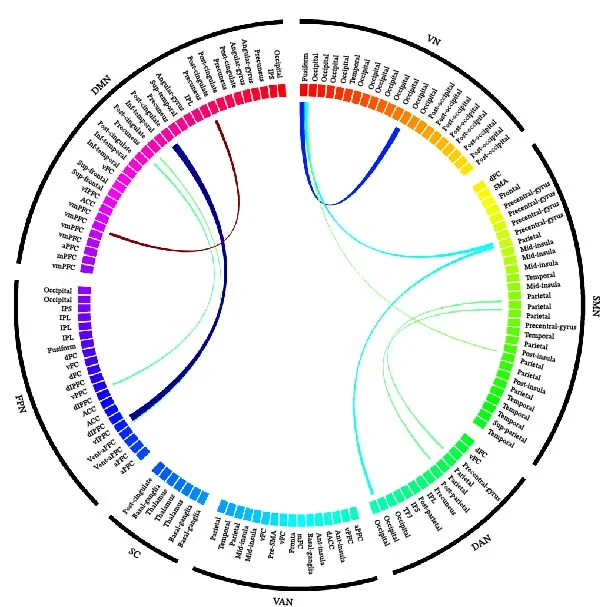
Circles plot of large‐scale network functional connectivity differences between DS and No DS groups. This circles plot illustrates the differences in large‐scale functional connectivity between major depressive disorder (MDD) patients with DS symptoms and those without (No DS). The plot highlights reduced functional connectivity between key regions. Each segment represents different brain networks: the dorsal attention network (DAN), default mode network (DMN), somatomotor network (SMN), ventral attention network (VAN), visual network (VN), and frontoparietal network (FPN). The inner circle contains individual rectangles, each representing a Dosenbach ROI, while the outer circle indicates the positions of these ROIs within the seven YEO networks: the DMN, VAN, VN, DAN, SMN, FPN, and SC. The figure illustrates differences in FC between the DS and No DS groups, with lines representing these differences. Thicker lines indicate higher *t*‐values. Covariates: age, sex, current episode duration, HAMD score, sedative‐hypnotic use, sedating‐antidepressant‐equivalent dose, and mean FD.

Large‐scale network FC analysis revealed widespread reductions in FC among MDD patients with DS symptoms compared to those without DS. These reductions were primarily clustered within two major topological domains (Figure 1). First, significant hypoconnectivity was observed between anterior prefrontal regions and the posterior medial core, notably involving connections from the vlPFC, ventromedial prefrontal cortex (vmPFC), and ventral prefrontal cortex (vPFC) to the PCC and precuneus. Second, diminished connectivity was identified within and between posterior parieto‐occipital and visual processing areas, encompassing intraparietal connections, as well as pathways linking the parietal lobe with the occipital lobe and fusiform gyrus and between the fusiform gyrus and occipital lobe.

**Figure 2:**
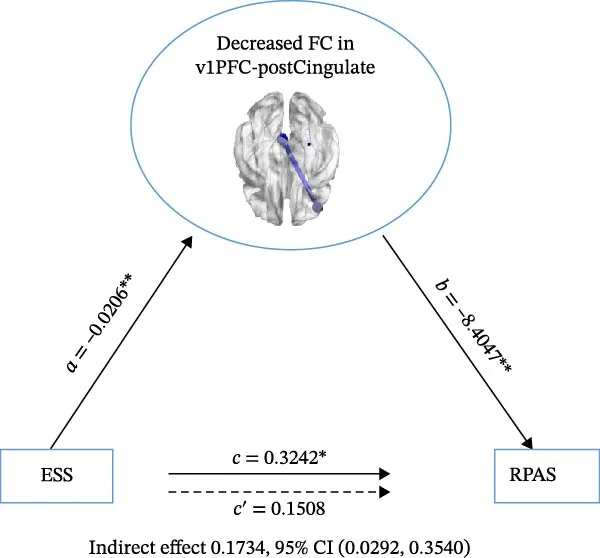
Mediation effect of functional connectivity between vlPFC and posterior cingulate cortex on the relationship between ESS and RPAS. The mediation analysis demonstrates the role of FC between the vlPFC and the posterior cingulate cortex in mediating the relationship between the Epworth Sleepiness Scale (ESS) and the Revised Physical Anhedonia Scale (RPAS). The diagram shows the pathways between ESS, vlPFC‐posterior cingulate connectivity, and RPAS, highlighting the indirect effects. Abbreviations: CI, confidence interval; ESS, Epworth Sleepiness Scale; PCC, posterior cingulate cortex; RPAS, Revised Physical Anhedonia Scale; vlPFC, ventrolateral prefrontal cortex. Covariates: age, sex, and mean FD. Symbols:  ^∗^ indicates *p* < 0.05, and  ^∗∗^ indicates *p* < 0.01.

To further investigate the clinical relevance of these network alterations, a mediation analysis was conducted. The analysis (PROCESS Model 4; 5000 BC bootstrap resamples; covariates: age, sex, and mean FD) demonstrated that vlPFC–PCC FC significantly mediated the association between ESS and RPAS (Figure 2).

### 3.3. Small‐World Properties

**Figure 3:**
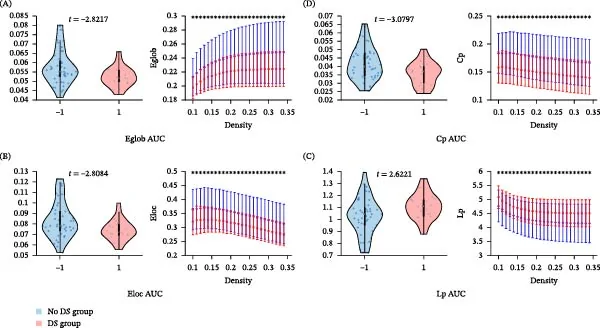
Between‐group differences in small‐world properties of functional brain networks. Between‐group differences in small‐world properties of functional brain networks across a wide range of density thresholds. The inserts represent the differences of the area under curve (AUC) for each topological parameter across the density range of 10% to about 34%. Each point and error bar denote the mean and standard deviation at each density level, respectively. Black asterisks indicate where the differences between groups are significant at a given density threshold (*t*‐test, *p*  < 0.05). Red represents the DS group, and blue represents the No DS group. The dotted lines in the line graphs indicate significant differences across the density range (*p*  < 0.05). (A) global efficiency (Eglob); (B) local efficiency (Eloc); (C) path length (Lp); and (D) clustering coefficient (Cp). Covariates: age, sex, current episode duration, HAMD score, sedative‐hypnotic use, sedating‐antidepressant‐equivalent dose, and mean FD.

Compared with MDD patients without DS symptoms, those with DS symptoms demonstrated reduced whole‐brain Eglob, Eloc, Cp, and prolonged Lp (*p* < 0.05, Figure 3). These differences were significant across the entire density range from 10% to 34%.

### 3.4. Sensitivity Analyses

All primary findings remained directionally consistent under three sets of alternative analyses (Supporting Information). (1) Nonparametric Mann–Whitney *U* tests confirmed the group difference in RPAS (*U* = 688, *p* = 0.010; Table S1). (2) Group comparisons of FC and graph‐theoretical metrics under a minimally adjusted model (covariates: age, sex, and mean FD) were directionally consistent with the primary fully‐adjusted model (Figures S3 and S4). (3) Reclassifying patients using the international cutoff ESS ≥ 10 (DS, *n* = 10; No DS, *n* = 57) confirmed the group difference in RPAS (*p* = 0.015) and additionally revealed group differences in RSAS and TEPS that were not detected at the ESS ≥ 7 threshold (Table S2); the latter findings should be interpreted with caution given the small DS subgroup (*n* = 10) and may reflect that more severe DS produces broader anhedonia phenotypes. Together, these analyses indicate that our central findings are robust to analytic choices.

## 4. Discussions

This study aimed to explore the relationship between DS and anhedonia in patients with MDD and to investigate the neuroimaging mechanisms using fMRI. The results showed that the DS group of MDD patients scored higher on the physical anhedonia scale and exhibited significant differences in brain network FC and whole‐brain network metrics. Specifically, FC between the DS group’s DMN and FPN mediated the relationship between sleepiness and anhedonia.

Previous studies have found that DS is one of the common symptoms in patients with MDD and is linked to poorer clinical outcomes. The results of this study are consistent with the research by Zhao et al. [30], who found that symptoms of DS in MDD patients are associated with more severe depressive symptoms [30]. Our study further confirms the association between symptoms of DS and the core symptom of depression in MDD patients: anhedonia.

We observed that MDD patients in the DS group had reduced FC between the vlPFC and the PCC, which may be related to the severity of anhedonia. The vlPFC appears to be a key node in the endogenous mu‐opioid system, as it is rich in mu‐opioid receptors compared to other prefrontal regions. This region has been previously implicated in anhedonia in MDD patients [31]. Additionally, improvements in cortical activity of the vlPFC after AD treatment have been found to be associated with improvements in anhedonia in MDD patients [32]. Studies have also found that the psycho–physiological interaction (PPI) between the dorsal medial prefrontal cortex (dmPFC) and PCC is associated with anhedonia in MDD patients [33], which aligns with the results of our study.

Furthermore, a previous study found that increased FC in the ventral medial prefrontal cortex of Parkinson’s disease patients with DS symptoms has been found to correlate with their ESS scores [14]. Although the population in this study differs from our own, it nonetheless underscores the significant role of FC within the PFC in relation to sleepiness symptoms. In our research, we observed that diminished FC between the vlPFC and the PCC correlates with both sleepiness symptoms and anhedonia in MDD patients. Moreover, this reduction in connectivity statistically mediates the association between these clinical manifestations.

Although our findings demonstrate that vlPFC–PCC FC mediates the association between DS and physical anhedonia, the cross‐sectional nature of our design warrants careful consideration of the underlying conceptual framework. Several nonmutually‐exclusive interpretations are possible. First, consistent with our a priori hypothesis, DS may exert downstream effects on physical anhedonia by reducing arousal‐dependent reward‐system engagement, with vlPFC–PCC connectivity as a key node in this pathway. Second, an alternative model is that anhedonia and DS may co‐occur as parallel manifestations of a shared dysfunction in DMN–FPN circuitry, particularly given that the vlPFC–PCC pair lies at the intersection of arousal‐regulating and reward‐evaluating networks. Third, reduced motivational engagement secondary to anhedonia could itself contribute to subjective sleepiness through behavioral disengagement. The cross‐sectional design of the present study cannot adjudicate among these competing models, and we present our mediation analysis as a test of one theoretically motivated pathway rather than as evidence of unique causal direction. Longitudinal and interventional studies will be needed to establish the temporal and causal relationships among DS, anhedonia, and DMN–FPN connectivity.

In adolescent narcolepsy, regional changes in the PCC have been found to be associated with subjective sleepiness and exhibit abnormalities in small‐world network properties [34]. In our study, we also found a reduction in the FC of the DMN and abnormalities in whole‐brain topological properties in patients with subjective sleepiness, which aligns with findings in adult MDD patients. Taken together, our findings indicate that DS is not merely a secondary concomitant symptom or a simple subjective complaint; rather, it represents a parallel clinical manifestation sharing a common neurobiological basis with anhedonia, reflecting dysfunction within large‐scale brain networks and warranting significant clinical attention. In particular, MDD patients with DS symptoms exhibited significant alterations in small‐world properties of brain networks, including reduced Eglob, Eloc, and Cp, as well as increased Lp. These changes suggest disruptions in the optimal organization of brain networks, potentially underlying the cognitive and emotional impairments observed in these patients.

Several limitations should be considered when interpreting our findings. First, the modest sample size (*n* = 67) limits statistical power, particularly for sensitivity analyses using stricter ESS cutoffs and for fully adjusted multivariable models in which collinearity among ESS, depression severity, and current episode duration constrained the available signal. Second, the cross‐sectional design precludes causal inference. Although our mediation analysis tests a specific theoretical pathway (DS → vlPFC–PCC → anhedonia), it cannot rule out alternative directional or shared‐substrate models, as discussed above. Future longitudinal and interventional studies will be required to establish temporal precedence among these constructs. Third, although we statistically adjusted for AD exposure (both total fluoxetine‐equivalent and sedating‐AD–equivalent doses) and sedative‐hypnotic use, residual medication–related confounding cannot be fully excluded, given that our patients were not drug‐naïve; the parallel direction of findings under multiple covariate sets nonetheless suggests robustness. Fourth, the absence of a healthy control group is a key limitation. Our study was specifically designed to examine within‐MDD heterogeneity according to DS status—analogous to studies stratifying MDD by melancholic vs. atypical features—but consequently, we cannot determine whether the observed neural alterations in the DS subgroup represent a deviation from, an accentuation of, or a different direction relative to a healthy baseline. Future studies should include both healthy controls and nondepressed individuals with DS to enable full disease‐by‐symptom dissociation. Fifth, the use of ESS ≥ 7 (rather than the international ≥ 10 threshold) was justified by population‐specific normative data; nevertheless, sensitivity analyses using ESS ≥ 10 yielded directionally consistent results. Future research should employ larger sample sizes and longitudinal designs to validate the findings of this study and explore the relationship between DS symptoms and other symptoms in MDD patients. Additionally, future studies could investigate the potential effects of various interventions, such as cognitive–behavioral therapy or pharmacological treatments, on alleviating DS symptoms and anhedonia. In addition, because participants were recruited from a single specialized sleep‐disorders department, the generalizability of our findings to community‐based MDD populations, other clinical settings, and individuals without MDD may be limited.

In conclusion, our study is the first to explicitly link DS symptoms with physical anhedonia in MDD patients, providing novel insights into their potential neural mechanisms. This pioneering work holds promise for refining the pathophysiological understanding of MDD. Future research should aim to corroborate these findings and explore targeted interventions to mitigate DS symptoms and anhedonia in MDD patients.

## Author Contributions

Dao‐min Zhu and Jiajia Zhu conceptualized and designed the study. Yifei Li was responsible for data collection, conducting the analyses, preparing the first draft of the manuscript, and preparing the manuscript for submission. Yu Zhang, Shukun Zhu, Qinghong Hu, and Peng Fang were responsible for data collection and initial data preprocessing. Dao‐min Zhu was responsible for obtaining funding for the study, supervising the analyses, and editing the drafts of the manuscript.

## Funding

This work was supported by the Key Scientific Research Project of Colleges and Universities of Anhui Province of China (Grant 2023AH050585), the Hefei Health Applied Medical Research Project (Grant Hwk2023zc003), the Seventh Cycle Medical Key Discipline (Sleep and Mental Health) of Hefei, the Key Specialty of Medical and Health Care (Psychiatry) of Anhui Province, and the National Key Clinical Specialty (Psychiatry).

## Disclosure

The funding sources provided financial and institutional support for this work. The study design, data collection, data analysis and interpretation, and manuscript preparation were conducted by the authors. All authors contributed to and approved the final manuscript. The final manuscript reflects the authors’ original intellectual contributions.

## Ethics Statement

The investigation was carried out in accordance with the latest version of the Declaration of Helsinki. The studies involving human participants were reviewed and approved by the Biomedical Ethics Committee of Anhui Medical University (Approval Number 832230466). informed consent of the participants was obtained after the nature of the procedures had been fully explained.

## Conflicts of Interest

The authors declare no conflicts of interest.

## Supporting Information

Additional supporting information can be found online in the Supporting Information section.

## Supporting information


**Supporting Information** The supporting information contains Tables S1–S2 and Figures S1–S4, including normality and nonparametric sensitivity analyses, analyses using the ESS ≥ 10 cutoff, a schematic overview of the study workflow, individual‐level RPAS distributions, and minimally adjusted functional–connectivity and graph‐theoretical analyses.

## Data Availability

The data that support the findings of this study are available from the corresponding author upon reasonable request.
