# Supplementary material for: Altered Functional Connectivity and Small‐World Network Properties in MDD With Daytime Sleepiness: Implications for Anhedonia
Source: Depress Anxiety. 2026 Sep 15;2026:9599586. doi: 10.1155/da/9599586 (PMC13575881; doi:10.1155/da/9599586)
Supplement: Supplementary file 1 [file DA-2026-9599586-s001.docx]

### **Supplementary Table S1:** **Normality and non-parametric tests**

| Variable | DS  Mean±SD | NoDS  Mean±SD | SW *p*  (DS) | SW *p*  (No-DS) | Levene's *p* | *t* | *p_t* | *U* | *p_U* |
| --- | --- | --- | --- | --- | --- | --- | --- | --- | --- |
| ESS | 10.14±4.11 | 2.31±2.22 | <0.001^***^ | <0.001^***^ | 0.350 | 10.14 | <0.001^***^ | 990 | <0.001^***^ |
| RPAS | 26.05±8.32 | 20.87±8.74 | 0.337 | 0.045 | 0.734 | 2.31 | 0.024^*^ | 688 | 0.010^*^ |
| RSAS | 21.41±7.81 | 19.80±6.17 | 0.664 | 0.124 | 0.170 | 0.92 | 0.362 | 556 | 0.414 |
| TEPS | 52.55±23.81 | 58.62±16.87 | 0.255 | 0.028 | 0.013 | -1.07 | 0.292 | 410 | 0.259 |
| age | 38.36±12.27 | 37.71±13.64 | 0.140 | <0.001^***^ | 0.574 | 0.19 | 0.850 | 512 | 0.820 |
| HAMD | 33.77±7.68 | 34.80±7.09 | 0.138 | 0.185 | 0.532 | -0.54 | 0.590 | 430 | 0.392 |
| HAMA | 22.41±7.81 | 21.13±5.88 | 0.072 | 0.001^**^ | 0.396 | 0.75 | 0.458 | 530 | 0.640 |
| AHI | 3.48±5.53 | 4.44±6.95 | <0.001^***^ | <0.001^***^ | 0.593 | -0.56 | 0.575 | 466 | 0.703 |
| AD_dose | 23.19±14.19 | 18.92±17.75 | 0.166 | <0.001^***^ | 0.353 | 0.99 | 0.328 | 589 | 0.202 |
| sed_AD_dose | 2.05±3.67 | 1.98±4.79 | <0.001^***^ | <0.001^***^ | 0.953 | 0.06 | 0.953 | 522 | 0.642 |
| FD | 0.058±0.026 | 0.060±0.037 | 0.003^**^ | <0.001^***^ | 0.736 | -0.25 | 0.801 | 499 | 0.963 |

### DS = daytime sleepiness group (ESS ≥ 7, n = 22); No-DS = group without daytime sleepiness (ESS < 7, n = 45). SW = Shapiro–Wilk test of normality (significant p indicates departure from normality). Levene's test is reported with median centering (Brown–Forsythe variant). Independent-samples t-tests assume equal variances; Mann–Whitney U tests are reported as a non-parametric sensitivity analysis. Bold *p*-values indicate violation of the corresponding assumption (SW, Levene's) or statistical significance at α = 0.05 (t, U). AD_dose = antidepressant fluoxetine-equivalent dose; sed_AD_dose = sedating-antidepressant-equivalent dose; FD = mean framewise displacement. * *p* < 0.05; ** *p*<0.01; *** *p* < 0.001.

### **Supplementary Table S2: Group Differences in Anhedonia Scales Using the ESS ≥ 10 Cutoff**

| Outcome | DS≥10  (n=10) | NoDS<10 (n=57) | *t* | *p_t* | *U* | *p_U* |
| --- | --- | --- | --- | --- | --- | --- |
| RPAS | 28.80±9.39 | 21.47±8.41 | 2.50 | 0.015 * | 433 | 0.009 * |
| RSAS | 25.10±6.52 | 19.49±6.47 | 2.53 | 0.014 * | 410 | 0.028 * |
| TEPS | 43.30±26.71 | 58.96±17.15 | -2.44 | 0.018 * | 135 | 0.009 * |

### Independent-samples t-tests (*t*, *p*_*t*) and Mann–Whitney U tests (*U*, *p_U*) comparing the three anhedonia scales between groups stratified by the international ESS ≥ 10 cutoff. RPAS = Revised Physical Anhedonia Scale; RSAS = Revised Social Anhedonia Scale; TEPS = Temporal Experience of Pleasure Scale. * *p* < 0.05.

### **Supplementary Figure S1:** **Schematic overview of the study workflow.**

### The flowchart illustrates the sequential phases of the study, including participant enrollment and screening, multimodal data acquisition (clinical scales, PSG, and rs-fMRI), group stratification based on the Epworth Sleepiness Scale (ESS), fMRI data processing pipelines, and the comprehensive statistical analysis framework. Abbreviations: MDD, Major Depressive Disorder; DS, Daytime Sleepiness; RPAS, Revised Physical Anhedonia Scale; RSAS, Revised Social Anhedonia Scale; TEPS, Temporal Experience of Pleasure Scale; HAMD, Hamilton Depression Rating Scale; HAMA, Hamilton Anxiety Rating Scale;

### **Supplementary Figure S2:** **Individual-level distribution of RPAS scores in MDD patients with and without daytime sleepiness**

### Bar plot with overlaid individual data points showing the distribution of Revised Physical Anhedonia Scale (RPAS) scores in the DS group (n=22) and No-DS group (n=45). The asterisk indicates statistical significance (independent-samples t-test, t = 2.314, p = 0.024). Error bars represent standard deviation.

### **Supplementary Figure S3:** **Group comparisons of resting-state functional connectivity using a minimally adjusted model**

This Circles Plot illustrates the differences in large-scale functional connectivity between MDD patients with daytime sleepiness (DS) and those without (No DS) under a minimally adjusted covariate model. Each segment represents a different brain network: the dorsal attention network (DAN), default mode network (DMN), somatomotor network (SMN), ventral attention network (VAN), visual network (VN), and frontoparietal network (FPN). The inner circle contains individual rectangles, each representing a Dosenbach ROI, while the outer circle indicates the positions of these ROIs within the seven Yeo networks (DMN, VAN, VN, DAN, SMN, FPN, and SC). Lines connecting ROIs represent significant between-group FC differences, with thicker lines indicating higher t-values. Compared with the fully-adjusted model presented in Figure 1, fewer connections survived under this minimally adjusted model; however, the surviving connections remained within the same network domains (DMN–FPN and posterior visual/parieto-occipital regions), indicating that the principal findings are directionally consistent across covariate sets. Covariates: age, sex, mean FD. Two-sample t-test, *p* < 0.05, FDR-corrected.

### **Supplementary Figure S4:** **Group comparisons of GTA analysis using a minimally adjusted model**

Between-group differences in small-world properties of functional brain networks across a wide range of density thresholds under a minimally adjusted covariate model. The inserts represent the differences of the area under the curve (AUC) for each topological parameter across the density range of 10% to approximately 34%. Each point and error bar denote the mean and standard deviation at each density level, respectively. Black asterisks indicate where the differences between groups are significant at a given density threshold (two-sample t-test, p < 0.05). Red represents the DS group, and blue represents the No-DS group. A) global efficiency (Eglob); B) local efficiency (Eloc); C) clustering coefficient (Cp); D) path length (Lp). The pattern of reduced Eglob, Eloc, and Cp and prolonged Lp in the DS group is directionally consistent with the fully-adjusted results presented in Figure 3. Covariates: age, sex, mean FD.
